# Classification and prediction for multi-cancer data with ultrahigh-dimensional gene expressions

**DOI:** 10.1371/journal.pone.0274440

**Published:** 2022-09-15

**Authors:** Li-Pang Chen

**Affiliations:** Department of Statistics, National Chengchi University, Taipei, Taiwan, ROC; Instituto Nacional de Medicina Genomica, MEXICO

## Abstract

Analysis of gene expression data is an attractive topic in the field of bioinformatics, and a typical application is to classify and predict individuals’ diseases or tumors by treating gene expression values as predictors. A primary challenge of this study comes from ultrahigh-dimensionality, which makes that (i) many predictors in the dataset might be non-informative, (ii) pairwise dependence structures possibly exist among high-dimensional predictors, yielding the network structure. While many supervised learning methods have been developed, it is expected that the prediction performance would be affected if impacts of ultrahigh-dimensionality were not carefully addressed. In this paper, we propose a new statistical learning algorithm to deal with multi-classification subject to ultrahigh-dimensional gene expressions. In the proposed algorithm, we employ the model-free feature screening method to retain informative gene expression values from ultrahigh-dimensional data, and then construct predictive models with network structures of selected gene expression accommodated. Different from existing supervised learning methods that build predictive models based on entire dataset, our approach is able to identify informative predictors and dependence structures for gene expression. Throughout analysis of a real dataset, we find that the proposed algorithm gives precise classification as well as accurate prediction, and outperforms some commonly used supervised learning methods.

## 1 Introduction

Analysis of gene expression data is an important topic in bioinformatics. A large body of research and relevant developments have been explored in recent years. One of important branches of gene expression data analysis is to take gene expression values as predictors to classify and predict tumors to possible cancers. A motivated example in this paper is the GCM dataset, which contains 16,063 gene expression values and 14 human cancers among 198 tumor samples. The goal of this study is to take gene expression values as the predictors, and use them to classify tumor samples to their corresponding cancers. In this dataset, a key feature is ultrahigh-dimensional predictors in the sense that the dimension of predictors (number of gene expression values) is extremely greater than the sample size (tumor samples). This feature further induces some challenges, including (a) pairwise interactions among gene expressions and (b) existence of non-informative gene expressions, that affect the performance of classification and the accuracy of prediction.

To address classification and prediction for biomedical research, many supervised learning methods have been developed and have been widely applied in machine learning frameworks. With the ignorance of pairwise interactions and existence of non-informative predictors induced by ultrahigh-dimensional predictors, [1] proposed the integration of several heterogeneous cancer series, and performed a multi-class classification. [2] studied multicategory support vector machine (SVM) for the classification of multiple cancer. [3] presented comprehensive discussions of SVM methods. [4] applied SVM ensembers to analyze breast cancer prediction. [5] discussed linear discrimination analysis (LDA) and its application in the microarray. [6] discussed the multi-class analysis by generalized sparse linear discriminant analysis. The detailed and fundamental discussions of those methods can be found in [7, 8], and were reviewed by [9] as well. In recent years, deep learning approaches, such as convolutional neural network (e.g., [10]) or natural language processing (e.g., [11]), have been developed to deal with multicalssification. More applications can be found in some monographs, such as [12–14].

To characterize pairwise interactions among gene expressions, which usually refers to the *network dependence* among gene expressions, we employ *graphical models* that are powerful methods in describing the dependence structure of variables. A general introduction of graphical models can be found in [7] (Chapter 17). In the past literature, graphical models have been used to deal with the classification problem. For example, [15] proposed the network-based support vector machine for the classification of microarray samples for binary classification. [16] discussed the identification of rheumatoid arthritis-related genes by using a network-based support vector machine. [17] proposed network linear discriminant analysis. [18] proposed the nearest neighbor network. Most existing methods focused on binary responses and restricted the predictors to follow the normal distribution because of explorations of the precision matrix. Furthermore, it is intuitive to understand that the network structure of variables in different classes may not be exactly equal to each other. To address this issue, [19, 20] explored SVM and logistic regressions with heterogeneous network structures accommodated, respectively. More recently, [21, 22] developed multiclass discriminant analysis with network structures accommodated. From the perspectives of Bayesian approaches, several methods were also investigated with the network structure incorporated, including [23, 24].

To address non-informative gene expression values in ultrahigh-dimensional data, variable selection or dimension reduction are perhaps commonly used strategies in the past literature. For example, [25] applied unsupervised feature extraction, such as principal component analysis, tensor decomposition, and kernel tensor decomposition, to select potentially important genes. [26] adopted SIS method to do feature screening for gene expressions and combined Nottingham Prognostic Index with a hybrid signature accommodated. With the combination of supervised learning, [27] proposed the penalized method for SVM. [28, 29] explored variable selection based on LDA. Those methods mainly handled the setting that the dimension is smaller than the sample size, however, it is unknown whether those methods are able to deal with the case that the dimension of predictors is much higher than the sample size.

**Fig 1:**
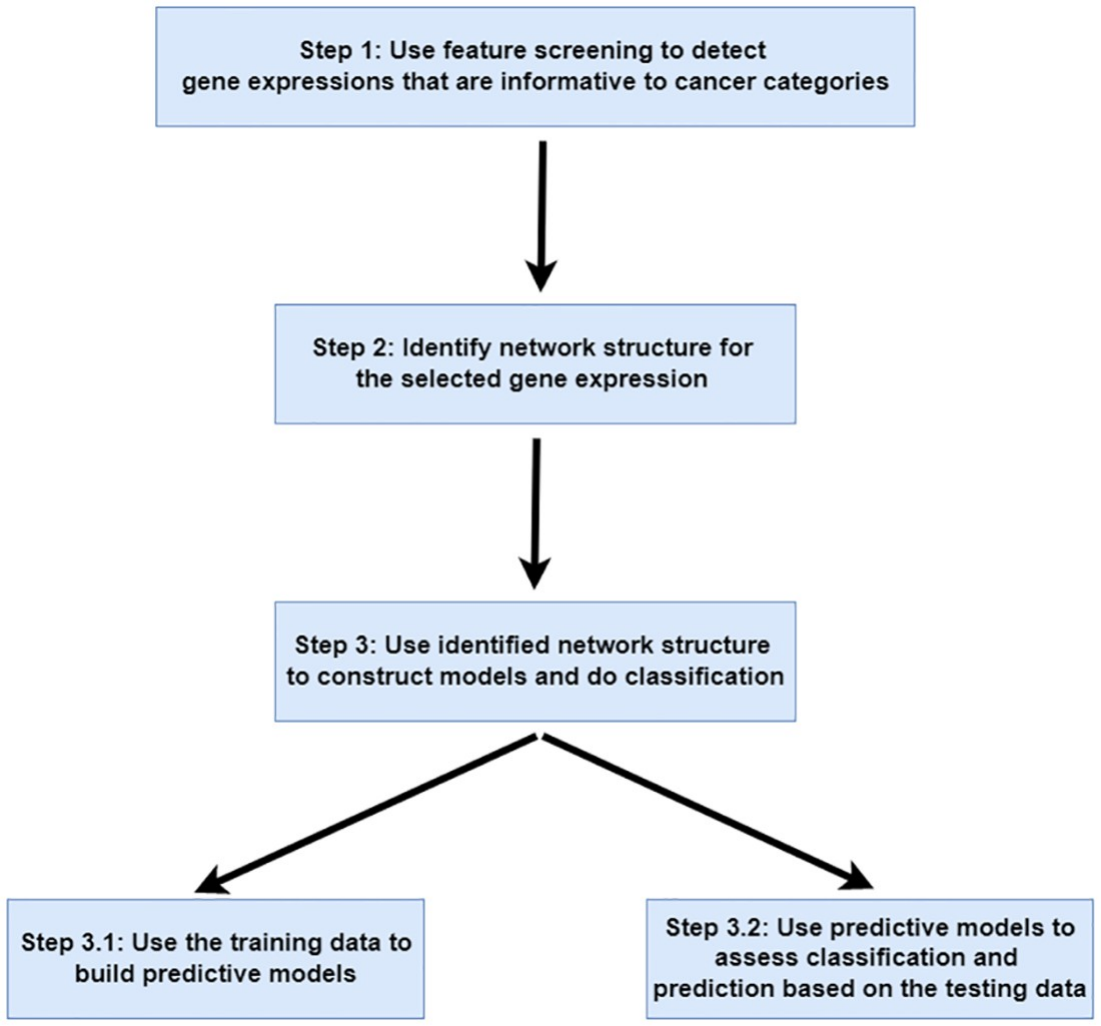
Summary of key steps for the proposed classification method via ultrahigh-dimensional gene expressions.

From the two challenges and developments described above, we note that most existing methods deal with either network structure or variable selection but not both. It motivates us to propose a strategy to *simultaneously* retain important predictors and construct the network structure of predictors when doing classification. Our strategy is outlined in Fig 1. Roughly speaking,

(i) to deal with ultrahigh-dimensional predictors where the dimension of predictors is extremely greater than the sample size, we adopt feature screening techniques to retain predictors that are informative to the response;
(ii) to detect network structures of predictors, we employ exponential family graphical models to detect network structure of the selected predictors under the whole dataset or different classes;
(iii) use the results in (i) and (ii) to develop network-based classification models to examine class separation and make the prediction for tumor samples.

There are several contributions in the proposed method. First, unlike existing methods that may specify a model when doing feature screening, our feature screening procedure is model-free and does not need to specify the model formulation. Second, although there exist methods handling network structures in classification, they assume a common network structure for predictors of all subjects without taking into account of possible heterogeneity for different classes. Instead, the proposed method is able to construct predictive models with possibly class-dependent network structures of predictors taken into account. Finally, the proposed method is able to handle multi-class labels with the accommodation of network structures in predictors, which is different from existing methods that either handle multiclassification but not use the information of network structure, or simply accommodate network structure to deal with binary classification.

The remainder is organized as following. In Section 2, we introduce a motivated real dataset and its data structure. In addition, we define the relevant mathematical notation. In Section 3, we give detailed presentation for each step in Fig 1. In Section 4, we implement the proposed method to analyze a real dataset and compare the proposed method with its competitors. A general discussion is presented in Section 5.

## 2 Data structure with multi-class responses

In this section, we first introduce a motivated dataset outlined in Section 1. After that, we define mathematical notation to describe the data structure with multi-class responses.

### 2.1 Description of motivated dataset

The data presented in the following are the GCM dataset collected by [30]. This dataset contains 16,063 gene expression values and 198 tumor samples, including 144 training samples (denoted as T) and 54 testing samples (denoted as V). In addition, 14 common human cancers, including Breast (BR), Prostate (PR), Lung (LU), Colorectal (CO), Lymphoma (LY), Bladder (BL), Melanoma (ML), Uterus (UT), Leukemia (LE), Renal (RE), Pancreas (PA), Ovarym (OV), Mesothelioma (ME) and CNS cancers, are included in the dataset. The sample sizes of each cancer are summarized in Table 1. Our main goal is to classify tumor samples into different categories of cancer according to gene expression values of the samples, which are treated as predictors.

**Table 1:** Sample sizes for each cancer. The first row with T contains sample sizes of the training data in cancer labels; the second row with V contains sample sizes of the testing data in cancer labels; the last row with “Total” contains sample sizes of the whole data in cancer labels.

	BR	PR	LE	CO	LU	BL	CNS	UT	LY	RE	PA	OV	ME	ML
T	8	8	8	8	16	8	8	8	24	8	8	8	8	16
V	3	4	2	4	4	4	2	3	6	4	3	6	3	6
Total	11	12	10	12	20	12	10	11	30	12	11	14	11	16

Even though this dataset is no need to pre-processing due to complete observations without missing value, and some of its features having been well analyzed by [30], still, the dataset can be further investigated in two aspects. First of all, we propose to note the issue of high-dimentionality of the data, which usually implies the existence of irrelevant variables, i.e., not every gene expression is dependent upon the response. Therefore, to ensure the accuracy of prediction, it is necessary to exclude irrelevant variables. As a result, it is crucial to select gene expressions that are informative in terms of responses. Secondly, as discussed in [31, 32], complex dependence structures may exist among high-dimensional gene expressions. Therefore, to increase the accuracy of predictions, it is necessary to incorporate the network structure of gene expressions into the classification procedure.

### 2.2 Notation

In this subsection, we define mathematical notation to describe the data in order to develop the method.

Suppose the data of *n* subjects come from *I* classes, where *I* is a fixed integer greater than 2 and the classes are nominal. Let *n*_*i*_ be the class size in class *i* with *i* = 1, ⋯, *I*, and hence $n=\sum_{i=1}^{I}n_{i}$. Let **Y** denote the *n*-dimensional vector of response with the *j*th component being *Y*_*j*_ = *i*, which reflects the class membership that the *j*th subject is in the *i*th class for *i* = 1, ⋯, *I* and *j* = 1, ⋯, *n*.

Let *p* > 1 denote the dimension of predictors for each subject. Define **X** = [*X*_*j*, *l*_] as the *n* × *p* matrix of predictors for *j* = 1, ⋯, *n* and *l* = 1, ⋯, *p*, where the component *X*_*j*,*l*_ represents the *l*th predictor for the *j*th subject. Furthermore, let *X*_*j*•_ = (*X*_*j*,1_, ⋯, *X*_*j*,*p*_)^⊤^ denote the *p*-dimensional predictor vector for the *j*th subject in the *j*th row of **X** and let *X*_•*k*_ = (*X*_1,*k*_, ⋯, *X*_*n*,*k*_)^⊤^ represent the *n*-dimensional vector of the *k*th predictor in the *k*th column of **X**. In this paper, we consider a setting that the dimension of the predictors *p* is ultrahigher than the sample size *n*, i.e., *p* = exp{*O*(*n*^*r*^)} for some constant *r* > 0 (e.g., [33]).

Without loss of generality, the {*X*_*j*•_, *Y*_*j*_} are treated as independent and identically distributed (i.i.d.) for *j* = 1, ⋯, *n*. We let lower case letters represent realized values for the corresponding random variables.

The objective of the study is to build models to predict the class label for a new subject with observation $\tilde{X}$.

## 3 Proposed method

In this section, we present detailed estimation procedure for each step as shown in Fig 1.

### 3.1 Feature screening via rank-based correlation coefficient

Let

$$I=\{k:X_{•k}\;\text{is}\;\text{dependent}\;\text{on}\;Y\in \left\{1,2,\cdots ,I\right\}\}$$

denote the *true active set* which contains all relevant predictors for the response *Y* with q=|I| and *q* < *n*, and $I^{c}$ is the complement of I that contains all irrelevant predictors for the response *Y*. Basically, the goal of Step 1 in Fig 1 is to estimate the active set I. When I is determined, then the associated vector of predictors $X_{I}=\{X_{•k}:k\in I\}$ contains important information in terms of the response, and its dimension is smaller than the sample size *n*. Thus, $X_{I}$ can be adopted to the subsequent analysis.

The remaining concern is to obtain the estimated active set. Following the spirit of [33], we employ the technique of feature screening, whose idea is to take the correlation of the response and the predictors as a signal, and retain the important predictors with large values of signals. We propose to take the rank-based correlation coefficient as the signal. Specifically, for the *k*th predictor *X*_•*k*_, the rank-based correlation coefficient between *X*_•*k*_ and *Y* is given by (e.g., [34, 35])

$$\begin{array}{c} \omega_{k}≜\xi \left(X_{•k},Y\right)=\frac{\int \text{var}[E\{I\left(Y\geq t\right)|X_{•k}\}]d\mu \left(t\right)}{\int \text{var}\{I(Y\geq t)\}d\mu \left(t\right)}, \end{array}$$ (1)

where I(·) denotes the indicator function and *μ*(⋅) is the law of *Y*. It can be shown that *ω*_*k*_ is in an interval [0, 1], and a higher value of *ω*_*k*_ indicates a stronger correlation between *Y* and *X*_•*k*_. Therefore, (1) can be regarded as similar to the classical coefficients such as Pearson’s correlation.

To implement this idea, we estimate (1) using the sample data. For *j* = 1, ⋯, *n*, denote *Y*_(*j*)_ as the rearranged response according to the sort of the *k*th predictors *X*_•*k*_, i.e., (*X*_(1),*k*_, *Y*_(1)_), ⋯, (*X*_(*n*),*k*_, *Y*_(*n*)_) with *X*_(1),*k*_ ≤ *X*_(2),*k*_ ≤ ⋯ ≤ *X*_(*n*),*k*_ and *X*_(*j*),*k*_ being the *j*th sorted predictor in *X*_•*k*_. The corresponding estimator of *ω*_*k*_ is given by [34]:

$$\begin{array}{c} {\hat{\omega }}_{k}≜\hat{\xi }\left(X_{•k},Y\right)=1-\frac{n\sum_{j=1}^{n-1}|r_{j+1}-r_{j}|}{2\sum_{j=1}^{n}\ell_{j}(n-\ell_{j})}, \end{array}$$ (2)

where, for *j* = 1, ⋯, *n*, $\ell_{j}≜\#\{l:Y_{\left(l\right)}\geq Y_{\left(j\right)}\}$, $r_{j}≜\#\{l:Y_{\left(l\right)}\leq Y_{\left(j\right)}\}$, and #A represents the number of elements in a set A. In applications, one can use the R package XICOR to compute (2).

Therefore, the estimated active set based on (2) is given by

$$\begin{array}{c} \hat{I}=\{k:{\hat{\omega }}_{k}\geq cn^{-\kappa }\;\text{for}\;k=1,\cdots ,p\}, \end{array}$$ (3)

where *c* and *κ* ∈ (0, 1/2) are prespecified threshold values. In applications, one can specify *c* and *κ* such that variables with the first $\left[\frac{n}{\text{log}\;n}\right]$ largest values of ${\hat{\omega }}_{k}$ can be retained, where [⋅] represents the ceiling function (e.g., [33, 35, 36]).

Different from the conventional feature screening method (e.g., [33]), the main advantage of (3) is *model-free feature screening* because it does not impose model formulation, and thus, (3) is able to detect predictors that may have nonlinear relationship with the response *Y*. Theoretically, by the similar derivations of [35], the *sure screening property* of (3) can be justified. That is, $P(I\subseteq \hat{I})\to 1$ as *n* → ∞, which ensures that the estimated active set contains truly informative predictors that are dependent on the response with a probability approaching one. Moreover, while there are several methods to deal with feature screening, as examined by [35], (2) generally outperforms other existing approaches and is able to handle oscillatory trajectory between the response and predictors.

When the active set is determined, we then let $X_{j,\hat{I}}=\{X_{j,k}:k\in \hat{I}\}$ denote the vector containing all the active predictors for the *j*th subject, and denote $x_{j,\hat{I}}$ as the realization values of $X_{j,\hat{I}}$.

### 3.2 The expressions of graphical structure

Since the estimated active set $\hat{I}$ is identified, we now explore the network structure of selected gene expressions in $\hat{I}$ for Step 2 in Fig 1. *Graphical models* are commonly used strategies to achieve this goal.

The graph is expressed as *G* = (*V*, *E*), where *V* is the set of the vertices and *E* ⊂ *V* × *V* is the set of the edges. In our case, $V≜\hat{I}$ is treated as selected predictors with $\tilde{q}=|V|$ and *E* is regarded as pairwise dependence of any two selected predictors. In graphical model frameworks, we start by formulating the distribution function of selected predictors. In this article, we consider exponential family graphical models because it generalizes the commonly used models. The formulation is given by

$$\begin{array}{ccc} P(X_{j,\hat{I}};\beta ,\Theta ) & = & \text{exp}\{\sum_{r\in V}\beta_{r}B\left(X_{j,r}\right)+\sum_{(s,t)\in E}\theta_{st}B\left(X_{j,s}\right)B\left(X_{j,t}\right)\\ & \;\; & +\sum_{r\in V}C\left(X_{j,r}\right)-A\left(\beta ,\Theta \right)\}, \end{array}$$ (4)

where $\beta =(\beta_{1},\cdots \beta_{\tilde{q}}{)}^{⊤}$ is the $\tilde{q}$-dimensional parameter vector, Θ = [*θ*_*st*_] is a $\tilde{q}\times \tilde{q}$ symmetric matrix, *B*(⋅) and *C*(⋅) are given functions that reflect the distribution of $X_{\hat{I}}$ (e.g., [20, 37]), and the function *A*(*β*, Θ) is normalizing constant which ensures (4) to be integrated as 1.

Without loss of general interest, we take *B*(*X*_*j*,*r*_) as the linear function *B*(*X*_*j*,*r*_) = *X*_*j*,*r*_ for *r* ∈ *V*. In addition, in the graphical model theory, the main interest is the estimation of *θ*_*st*_ because of its interpretation that *X*_*j*,*s*_ and *X*_*j*,*t*_ are conditionally dependent if *θ*_*st*_ ≠ 0. Therefore, to focus on presenting the estimation of *θ*_*st*_, we drop the main effect term, and consider the following graphical model

$$\begin{array}{c} P\left(X_{j,\hat{I}};\Theta \right)=\text{exp}\{\sum_{(s,t)\in E}\theta_{st}X_{j,s}X_{j,t}+\sum_{r\in V}C\left(X_{j,r}\right)-A\left(\Theta \right)\}, \end{array}$$ (5)

where the function *A*(Θ) is normalization constant which makes (5) be integrated as 1.

For the estimation method for Θ, one of the famous methods is the conditional inference [38]. Without loss of generality, we consider the vertex *s*, and define the *neighbourhood set*

$$\begin{array}{c} N\left(s\right)=\{t\in V:(s,t)\in E\}, \end{array}$$ (6)

which collect vertexes that are dependent on the vertex *s*. To estimate the neighbourhood set of *s*, it suffices to study the inference of *X*_*j*,*s*_|*X*_*j*,*V*\{*s*}_, where $X_{j,V\backslash \{s\}}=\left(X_{j,1},\cdots ,X_{j,s-1},X_{j,s+1},\cdots ,X_{j,\tilde{q}}\right)$. Let $\theta_{s}=(\theta_{s1},\cdots ,\theta_{s(s-1)},\theta_{s(s+1)},\cdots ,\theta_{s\tilde{q}})$ denote the $\left(\tilde{q}-1\right)$-dimensional vector of parameters that is associated with *X*_*j*,*V*\{*s*}_. By some algebra, we have

$$\begin{array}{ccc} P(X_{j,s}|X_{j,V\backslash \{s\}};\theta_{s}) & \propto & \text{exp}\{X_{j,s}(\sum_{t\in V\backslash \{s\}}\theta_{st}X_{j,t})+C(X_{j,s})\\ & \;\; & -D(\sum_{t\in V\backslash \{s\}}\theta_{st}X_{j,t})\}, \end{array}$$ (7)

where *D*(⋅) is a normalization constant ensuring that the integration of (7) is equal to 1. Then the estimator of *θ*_*s*_, denoted as ${\hat{\theta }}_{s}$, is given by

$$\begin{array}{c} {\hat{\theta }}_{s}=\underset{\theta_{s}}{\text{argmin}}\{\ell (\theta_{s})+\lambda \|\theta_{s}{\|}_{1}\}, \end{array}$$ (8)

where

$$\begin{array}{c} \ell (\theta_{s})=\frac{1}{n}\sum_{i=1}^{n}\{-X_{i,s}(\sum_{t\in V\backslash \{s\}}\theta_{st}X_{i,t})\\ +D(\sum_{t\in V\backslash \{s\}}\theta_{st}X_{i,t})\}, \end{array}$$

‖⋅‖_1_ is the *L*_1_-norm and λ is the tuning parameter.

In the penalization problem for selecting the variables, estimating the tuning parameter is also a crucial issue. In this paper, we employ the BIC approach (e.g., [39]) to select the tuning parameter λ. To emphasize the dependence on the tuning parameter, we let ${\hat{\theta }}_{s}\left(\lambda \right)$ denote the estimator obtained from (8). Define

$$\begin{array}{c} \text{BIC}(\lambda )=2n\ell ({\hat{\theta }}_{s}\left(\lambda \right))+\text{log}\left(n\right)\times \text{df}\{{\hat{\theta }}_{s}\left(\lambda \right)\}, \end{array}$$ (9)

where $\text{df}\{{\hat{\theta }}_{s}\left(\lambda \right)\}$ represents the number of non-zero elements in ${\hat{\theta }}_{s}\left(\lambda \right)$ for a given λ. The optimal tuning parameter λ, denoted by $\hat{\lambda }$, is determined by minimizing (9) within suitable ranges of λ. As a result, the estimator of *θ*_*s*_ is determined by ${\hat{\theta }}_{s}={\hat{\theta }}_{s}(\hat{\lambda })$.

Finally, the estimated neighbourhood set is given by

$$\begin{array}{c} \hat{N}\left(s\right)=\{t\in V:{\hat{\theta }}_{st}\neq 0\}. \end{array}$$ (10)

Note that *θ*_*st*_ is equal to *θ*_*ts*_ since Θ is a symmetric matrix. However, the estimators ${\hat{\theta }}_{st}$ and ${\hat{\theta }}_{ts}$ are not equal. To overcome this problem, we apply the AND rule [38], which indicates that the final estimators of ${\hat{\theta }}_{st}$ and ${\hat{\theta }}_{ts}$ are determined by their maximum if both ${\hat{\theta }}_{st}$ and ${\hat{\theta }}_{ts}$ are nonzero; ${\hat{\theta }}_{st}$ and ${\hat{\theta }}_{ts}$ are set to be zero if one of them is zero. Moreover, the estimated set of edges is given by

$$\begin{array}{c} \hat{E}=\{\left(s,t\right):s\in \hat{N}\left(t\right)\;\text{and}\;t\in \hat{N}\left(s\right)\}. \end{array}$$ (11)

After deriving the estimated set of edges, a crucial question is the relationship of $\hat{E}$ and *E*. To answer this question, we present the following theorem, which gives an important result for the estimated graph.

**Theorem 3.1** (*Network Recovery*)

*Suppose E is the set of edges, and let* $\hat{E}$ *be the estimated set of edges. Under some regular conditions in* [38], *we have that as n* → ∞,

$$\begin{array}{c} P(\hat{E}=E)\to 1. \end{array}$$ (12)

This result and regular conditions are similar to Section 2.2 in [40] and Theorem 5 (b) in [37]. Theorem 3.1 tells us that based on the mild conditions, the estimated network structure can be recovered to the true network structure.

### 3.3 Multinomial logistic regression with homogeneous network structure in predictors

After obtaining the estimated network structure based on informative predictors, we wish to use such a network structure to examine the classification for different cancers, as demonstrated in Step 3 of Fig 1. Therefore, to incorporate the network structures of the predictors into a prediction model, we present two methods which can be readily implemented using the R package glm for fitting a logistic regression model.

In the first method, called the *multinomial logistic regression with homogeneous network structure in predictors* (MLR-HomoNet), we consider the case where the subjects in different classes share a common network structure in the predictors. To build a prediction model, we make use of the development of the logistic model with multiclass responses ([41], Section 6.1; [42], Section 7.1).

We first identify the pairwise dependence of the predictors using the measurements of all the subjects without distinguishing their class label. Let ${\hat{\theta }}_{st}$ be the estimate for *θ*_*st*_ obtained for (8) by using all the predictor measurements of $\left\{X_{j,\hat{I}}:j=1,\cdots ,n\right\}$, and let $\hat{E}=\{\left(s,t\right):{\hat{\theta }}_{st}\neq 0\}$ denote the resulting estimated set of edges.

Next, for *i* = 1, ⋯, *I* and *j* = 1, ⋯, *n*, we let

$$p_{i}\left(x_{j,\hat{I}}\right)≜P(Y_{j}=i|X_{j,\hat{I}}=x_{j,\hat{I}})$$

be the conditional probability of *Y*_*j*_ = *i* given $X_{j,\hat{I}}=x_{j,\hat{I}}$. Consider the parametric multinomial logistic model

$$\begin{array}{c} p_{i}\left(x_{j,\hat{I}}\right)≜p_{i}\left(x_{j,\hat{I}};\alpha \right)=\frac{\text{exp}(\alpha_{i0}+\sum_{\left(s,t\right)\in \hat{E}}\alpha_{i,st}x_{j,s}x_{j,t})}{1+\sum_{l=1}^{I-1}\text{exp}(\alpha_{l0}+\sum_{\left(s,t\right)\in \hat{E}}\alpha_{l,st}x_{j,s}x_{j,t})} \end{array}$$ (13)

for *i* = 1, 2, ⋯, *I* − 1, where $\alpha =(\alpha_{1}^{⊤},\cdots ,\alpha_{I-1}^{⊤}{)}^{⊤}$ is the vector of parameters with vectors $\alpha_{i}≜(\alpha_{i0},\alpha_{i•}^{⊤}{)}^{⊤}$ and $\alpha_{i•}=(\alpha_{i,st}:\left(s,t\right)\in \hat{E}{)}^{⊤}$ reflecting parameters for class *i*, and the constraint $\sum_{i=1}^{I}p_{i}\left(x_{j,\hat{I}}\right)=1$ is imposed for every *j* = 1, ⋯, *n*.

For subject *j* = 1, ⋯, *n*, we let $Y_{ij}^{*}=1$ if subject *j* is in class *i* and $Y_{ij}^{*}=0$ otherwise, and hence, $\sum_{i=1}^{I}Y_{ij}^{*}=1$ for every *j*. Let $y_{ij}^{*}$ denote a realized value of $Y_{ij}^{*}$. For *i* = 1, ⋯, *I* and *j* = 1, ⋯, *n*, the log-likelihood function is given by ([42], p.273)

$$\begin{array}{c} L\left(\alpha \right)=\sum_{i=1}^{I}\sum_{j=1}^{n}y_{ij}^{*}\;\text{log}\{p_{i}\left(x_{j,\hat{I}};\alpha \right)\}. \end{array}$$ (14)

The estimator of *α*, denoted $\hat{\alpha }$, can be derived by maximizing (14). In applications, since $\hat{\alpha }$ has no closed form, we usually implement the Newton-Raphson algorithm to (14) and obtain the resulting estimator. Therefore, for the realization $x_{j,\hat{I}}$ of the *q*-dimensional vector $X_{j,\hat{I}}$, $p_{i}\left(x_{j,\hat{I}}\right)$ is estimated as

$$\begin{array}{c} {\hat{p}}_{i}\left(x_{j,\hat{I}}\right)≜p_{i}\left(x_{j,\hat{I}};\hat{\alpha }\right)=\frac{\text{exp}({\hat{\alpha }}_{i0}+\sum_{\left(s,t\right)\in \hat{E}}{\hat{\alpha }}_{i,st}x_{j,s}x_{j,t})}{1+\sum_{l=1}^{I-1}\text{exp}({\hat{\alpha }}_{l0}+\sum_{\left(s,t\right)\in \hat{E}}{\hat{\alpha }}_{l,st}x_{j,s}x_{j,t})} \end{array}$$ (15)

for *i* = 1, ⋯, *I* − 1, and $p_{I}\left(x_{j,\hat{I}}\right)$ is estimated as

$$\begin{array}{c} {\hat{p}}_{I}\left(x_{j,\hat{I}}\right)=1-\sum_{i=1}^{I-1}{\hat{p}}_{i}\left(x_{j,\hat{I}}\right). \end{array}$$ (16)

Finally, to predict the class label for a new subject with a selected $\tilde{q}$-dimensional predictor instance $\tilde{x}$, we first calculate the right-hand side of (15) and (16), and let ${\tilde{\hat{p}}}_{1},\cdots ,{\tilde{\hat{p}}}_{I}$ denote the corresponding values. Let i* denote the index which corresponds to the largest value of $\left\{{\tilde{\hat{p}}}_{1},\cdots ,{\tilde{\hat{p}}}_{I}\right\}$, i.e., $i^{*}=\underset{i\in \{1,\cdots ,I\}}{\text{argmax}}\;{\tilde{\hat{p}}}_{i}$. Then the class label for this new subject is predicted as i*.

To the end, we summarize key steps in Sections 3.1–3.3 in Algorithm 1.

**Algorithm 1**: MLR-HomoNet

Under the training data T;

**Step 1**: Determine informative predictors

  Apply (2) to do feature screening and retain $\left[\frac{n}{\text{log}\;n}\right]$ predictors among *p*-dimensional predictors. A set of selected predictors is given by (3).

**Step 2**: Determine the network structure of predictors

  Based on selected predictors in $\hat{I}$, use (8) to determine pairwise dependence structure and obtain (11). The resulting network structure is formed by $\hat{E}$.

**Step 3**: Construct the predictive model

  Given a initial value *α*^(0)^, then perform the following Newton-Raphson algorithm;

  **for** *step t with t* = 1, 2, ⋯, *T*, *say T* = 1000 **do**

 Step 3.1: calculate the score function evaluated at the *t*th iterated value:

$$S\left(\alpha^{\left(t\right)}\right)≜(\frac{\partial L(\alpha )}{\partial \alpha_{1}}{|}_{\alpha =\alpha^{\left(t\right)}},\cdots ,\frac{\partial L(\alpha )}{\partial \alpha_{I-1}}{|}_{\alpha =\alpha^{\left(t\right)}}{)}^{⊤}$$

 with

$$\frac{\partial L(\alpha )}{\partial \alpha_{i}}{|}_{\alpha =\alpha^{\left(t\right)}}=\sum_{j=1}^{n}\{y_{ij}(x_{j,\hat{I}}-p_{i}\left(x_{j,\hat{I}};\alpha^{\left(t\right)}\right))-y_{Ij}p_{i}\left(x_{j,\hat{I}};\alpha^{\left(t\right)}\right)\}.$$

 Step 3.2: calculate the Henssian matrix evaluated at the *t*th iterated value:

$$H\left(\alpha^{\left(t\right)}\right)≜\text{diag}(\frac{\partial^{2}L\left(\alpha \right)}{\partial \alpha_{1}\partial \alpha_{1}^{⊤}}{|}_{\alpha =\alpha^{\left(t\right)}},\cdots ,\frac{\partial^{2}L\left(\alpha \right)}{\partial \alpha_{I-1}\partial \alpha_{I-1}^{⊤}}{|}_{\alpha =\alpha^{\left(t\right)}})$$

 with

$$\frac{\partial^{2}L\left(\alpha \right)}{\partial \alpha_{i}\partial \alpha_{i}^{⊤}}{|}_{\alpha =\alpha^{\left(t\right)}}=-\sum_{j=1}^{n}\left(y_{ij}-y_{Ij}\right)p_{i}\left(x_{j,\hat{I}};\alpha^{\left(t\right)}\right)\{1-p_{i}\left(x_{j,\hat{I}};\alpha^{\left(t\right)}\right)\}.$$

 Step 3.3: update *α*^(*t*+1)^ ← *α*^(*t*)^ − {*H*(*α*^(*t*)^)}^−1^ *S*(*α*^(*t*)^);

  **end**

  Let $\hat{\alpha }≜\alpha^{\left(T\right)}$ denote the resulting estimator, and combine $\hat{\alpha }$ with (15) and (16) to determine the resulting predictive model ${\hat{p}}_{i}\left(x\right)$ for *i* = 1, ⋯, *I*.

Under the testing data V;

**Step 4**: Prediction

  For a new predictor $\tilde{x}$ in V, use ${\hat{p}}_{i}\left(x\right)$ with *i* = 1, ⋯, *I* to compute the corresponding probabilities ${\tilde{\hat{p}}}_{1},\cdots ,{\tilde{\hat{p}}}_{I}$. The predicted class i* is then determined by $i^{*}=\underset{i\in \{1,\cdots ,I\}}{\text{argmax}}\;{\tilde{\hat{p}}}_{i}$.

### 3.4 Logistic regression with heterogeneous network structured in predictors

We now present an alternative method to that described in Section 3.3. Instead of pooling all the predictors to feature the predictor network structure, this method, called the *logistic regression with heterogeneous network structured in predictors* (LR-HeteNet), stratifies the predictor information by class when characterizing the predictor network structures. The implementation is summarized in Algorithm 2.

**Algorithm 2**: LR-HeteNet

Under the training data T;

**for** *i* = 1, 2, ⋯, *I* **do**

  **Step 0**: Let *Y*^*i*^ denote an *n*-dimensional vector formulated by (17).

  **Step 1**: Class-dependent active set

    Apply (18) to do feature screening and retain $\left[\frac{n_{i}}{\text{log}\;n_{i}}\right]$ predictors among *p*-dimensional predictors. A set of selected predictors for class *i* is given by (19).

  **Step 2**: Class-dependent predictor network

    Based on selected predictors in ${\hat{J}}_{i}$, use (8) to determine pairwise dependence structure and obtain (11). Denote ${\hat{E}}^{i}$ as the resulting network structure.

  **Step 3**: Class-dependent predictive model

    Given a initial value $\gamma_{i}^{\left(0\right)}$, then perform the Newton-Raphson algorithm;

    **for** *step t with t* = 1, 2, ⋯, *T*, *say T* = 1000 **do**

   Step 3.1: calculate the score function evaluated at the *t*th iterated value:

$$S_{i}\left(\gamma_{i,(t)}\right)≜\frac{\partial L_{i}\left(\gamma_{i}\right)}{\partial \gamma_{i}}{|}_{\gamma_{i}=\gamma_{i}^{\left(t\right)}}=\sum_{j=1}^{n_{i}}x_{j,{\hat{J}}_{i}}\{y_{j}^{i}-\pi_{i}\left(x_{j,{\hat{J}}_{i}};\gamma_{i}^{\left(t\right)}\right)\},$$

   where $\pi_{i}\left(x_{j,{\hat{J}}_{i}};\gamma_{i}^{\left(t\right)}\right)$ is (20) with parameters replaced by $\gamma_{i}^{\left(t\right)}$;

   Step 3.2: calculate the Henssian matrix evaluated at the *t*th iterated value:

$$H_{i}\left(\gamma_{i}^{\left(t\right)}\right)≜\frac{\partial^{2}L_{i}\left(\gamma_{i}\right)}{\partial \gamma_{i}\partial \gamma_{i}^{⊤}}{|}_{\gamma_{i}=\gamma_{i}^{\left(t\right)}}=\sum_{j=1}^{n_{i}}x_{j,{\hat{J}}_{i}}x_{j,{\hat{J}}_{i}}^{⊤}\pi_{i}\left(x_{j,{\hat{J}}_{i}};\gamma_{i}^{\left(t\right)}\right)\{1-\pi_{i}\left(x_{j,{\hat{J}}_{i}};\gamma_{i}^{\left(t\right)}\right)\}.$$

   Step 3.3: update $\gamma_{i}^{\left(t+1\right)}\leftarrow \gamma_{i}^{\left(t\right)}-\{H_{i}\left(\gamma_{i}^{\left(t\right)}\right){\}}^{-1}S_{i}\left(\gamma_{i}^{\left(t\right)}\right)$;

    **end**

    Let $\hat{\gamma }≜\gamma_{i}^{\left(T\right)}$ denote the resulting estimator, and combine $\hat{\gamma }$ and (22) to determine the resulting predictive model ${\hat{\pi }}_{i}\left(x\right)$.

**end**

Under the testing data V;

**Step 4**: Prediction

    For a new predictor $\tilde{x}$ in V, we use ${\hat{\pi }}_{i}\left(x\right)$ with *i* = 1, ⋯, *I* to compute the corresponding probabilities ${\tilde{\hat{\pi }}}_{1},\cdots ,{\tilde{\hat{\pi }}}_{I}$. The predicted class i* is then determined by $i^{*}=\underset{i\in \{1,\cdots ,I\}}{\text{argmax}}\;\tilde{{\hat{\pi }}_{i}}$.

Be more specific, under the training data T, we first introduce a binary, surrogate response variable for every *i* = 1, ⋯, *I* and *j* = 1, ⋯, *n*. Let

$$\begin{array}{c} Y_{j}^{i}=\{\begin{array}{cc} 1, & Y_{j}=i\\ 0, & \text{otherwise}, \end{array} \end{array}$$ (17)

and let $Y^{i}=(0,\cdots ,0,Y_{1}^{i},\cdots ,Y_{n_{i}}^{i},0,\cdots ,0{)}^{⊤}$ be an *n*-dimensional vector whose elements corresponding to class *i* are respectively $Y_{1}^{i},\cdots ,Y_{n_{i}}^{i}$, and the other elements are zero. That is, $Y^{i}={\left(\underset{n_{1}+\cdots +n_{i-1}}{\underset{︸}{0,\cdots ,0}},\underset{n_{i}}{\underset{︸}{1,\cdots ,1}},\underset{n_{i+1}+\cdots +n_{I}}{\underset{︸}{0,\cdots ,0}}\right)}^{⊤}$ with *i* = 1, ⋯, *I*.

After that, we adopt the similar strategy in Algorithm 1 to construct predictive models for class *i*. Specifically, in Step 1 of Algorithm 2, let

$$J_{i}=\{k:X_{•k}\;\text{is}\;\text{dependent}\;\text{on}\;Y^{i}\}$$

denote the true active set of the class *i* which contains all relevant predictors for the response *Y*^*i*^ with $|J_{i}|<n_{i}$. Following (2), the signal of *X*_•*k*_ and *Y*^*i*^ is defined as $\omega_{k}^{i}≜\xi \left(X_{•k},Y^{i}\right)$, and it can be estimated by

$$\begin{array}{c} {\hat{\omega }}_{k}^{i}≜\hat{\xi }\left(X_{•k},Y^{i}\right)=1-\frac{n\sum_{j=1}^{n-1}|r_{j+1}^{i}-r_{j}^{i}|}{2\sum_{j=1}^{n}\ell_{j}^{i}(n-\ell_{j}^{i})}, \end{array}$$ (18)

where, for *j* = 1, ⋯, *n*, $\ell_{j}^{i}≜\#\{l:Y_{\left(l\right)}^{i}\geq Y_{\left(j\right)}^{i}\}$ and $r_{j}^{i}≜\#\{l:Y_{\left(l\right)}^{i}\leq Y_{\left(j\right)}^{i}\}$ with $Y_{\left(j\right)}^{i}$ being the rearranged response according to the sort of the *k*th predictors *X*_•*k*_. Therefore, $J_{i}$ can be estimated as

$$\begin{array}{c} {\hat{J}}_{i}=\{k:{\hat{\omega }}_{k}^{i}\geq c_{i}n^{-\kappa_{i}}\;\text{for}\;k=1,\cdots ,p\}, \end{array}$$ (19)

where *c*_*i*_ and *κ*_*i*_ ∈ (0, 1/2) are some prespecified threshold values. Let $X_{j,{\hat{J}}_{i}}=\{X_{j,k}:k\in {\hat{J}}_{i}\}$ denote the vector of all the active predictors that depends on *Y*^*i*^ for the *j*th subject. Moreover, since *Y*^*i*^ is defined as the response with binary outcomes, similar derivations in [35] show that (18) is valid to measure the dependence between categorical and continuous variables, and the point-biserial correlation coefficient is a special case of (18).

In Step 2 of Algorithm 2, let $V^{i}≜{\hat{J}}_{i}$ denote the vertex set containing predictors that are dependent on the class *i* = 1, ⋯, *I*. We apply the procedure described in Section 3.2 to determine the network structure of predictors in the class *i*. Let ${\hat{E}}^{i}=\{\left(s,t\right):{\hat{\theta }}_{st}^{i}\neq 0\}$ denote an estimated set of edges for the class *i*, where ${\hat{\theta }}_{st}^{i}$ is the estimate of *θ*_*st*_ derived from (8) based on using the predictor measurements in the class *i*.

After that, Step 3 in algorithm 2 aims to fit a logistic regression model using the surrogate response vector *Y*^*i*^ with the estimated predictors network structure ${\hat{E}}^{i}$ incorporated for *i* = 1, ⋯, *I*. Specifically, for the *j*th component of *Y*^*i*^, say $Y_{j}^{i}$, define $\pi_{i}\left(x_{j,{\hat{J}}_{i}}\right)=P(Y_{j}^{i}=1|X_{j,{\hat{J}}_{i}}=x_{j,{\hat{J}}_{i}})$ and consider the parametric logistic regression model

$$\begin{array}{c} \pi_{i}\left(x_{j,{\hat{J}}_{i}}\right)≜\pi_{i}\left(x_{j,{\hat{J}}_{i}};\gamma_{i}\right)=\frac{\text{exp}(\gamma_{i0}+\sum_{\left(s,t\right)\in {\hat{E}}^{i}}\gamma_{i,st}x_{j,s}x_{j,t})}{1+\text{exp}(\gamma_{i0}+\sum_{\left(s,t\right)\in {\hat{E}}^{i}}\gamma_{i,st}x_{j,s}x_{j,t})}, \end{array}$$ (20)

where *j* = 1, ⋯, *n*, $\gamma_{i}≜(\gamma_{i0},\gamma_{i•}^{⊤})$ with $\gamma_{i•}=(\gamma_{i,st}:\left(s,t\right)\in {\hat{E}}^{i}{)}^{⊤}$ is the vector of parameters associated with class *i*. In the spirit of the maximum likelihood estimation (MLE) method (e.g., [42]), the log-likelihood function of (20) is given by

$$\begin{array}{c} L_{i}\left(\gamma_{i}\right)=\sum_{j=1}^{n_{i}}[y_{j}^{i}\pi_{i}\left(x_{j,{\hat{J}}_{i}};\gamma_{i}\right)+\left(1-y_{j}^{i}\right)\{1-\pi_{i}\left(x_{j,{\hat{J}}_{i}};\gamma_{i}\right)\}], \end{array}$$ (21)

and the estimator of *γ*_*i*_, denoted ${\hat{\gamma }}_{i}≜({\hat{\gamma }}_{i0},{\hat{\gamma }}_{i•}^{⊤})$, is obtained by maximizing (21). In applications, we implement the Newton-Raphson algorithm to obtain ${\hat{\gamma }}_{i}$; the detailed procedure is summarized in Algorithm 2. Consequently, for the realization $x_{j,{\hat{J}}_{i}}$ of the $|{\hat{J}}_{i}|$-dimensional vector $X_{j,{\hat{J}}_{i}}$, based on (20), $\pi_{i}\left(x_{j,{\hat{J}}_{i}}\right)$ can be estimated by

$$\begin{array}{c} {\hat{\pi }}_{i}\left(x_{j,{\hat{J}}_{i}}\right)≜\pi_{i}\left(x_{j,{\hat{J}}_{i}};{\hat{\gamma }}_{i}\right)=\frac{\text{exp}({\hat{\gamma }}_{i0}+\sum_{\left(s,t\right)\in {\hat{E}}^{i}}{\hat{\gamma }}_{i,st}x_{j,s}x_{j,t})}{1+\text{exp}({\hat{\gamma }}_{i0}+\sum_{\left(s,t\right)\in {\hat{E}}^{i}}{\hat{\gamma }}_{i,st}x_{j,s}x_{j,t})} \end{array}$$ (22)

for *i* = 1, ⋯, *I*.

Finally, when predictive models based on the training data T are obtained, we now examine the prediction for the testing data V in Step 4 of Algorithm 2. Let ${\tilde{x}}_{j,{\hat{J}}_{i}}$ denote a $|{\hat{J}}_{i}|$-dimensional predictor vector for a new subject. We calculate (22) with $x_{j,{\hat{J}}_{i}}$ replaced by ${\tilde{x}}_{j,{\hat{J}}_{i}}$ for *i* = 1, ⋯, *I*, and let ${\tilde{\hat{\pi }}}_{1},\cdots ,{\tilde{\hat{\pi }}}_{I}$ denote the corresponding values. Let i* denote the index which corresponds to the largest value of $\left\{{\tilde{\hat{\pi }}}_{1},\cdots ,{\tilde{\hat{\pi }}}_{I}\right\}$, i.e.,

$$\begin{array}{c} {\tilde{\hat{\pi }}}_{i^{*}}=\underset{i\in \{1,\cdots ,I\}}{\text{max}}\tilde{{\hat{\pi }}_{i}}. \end{array}$$ (23)

Then the class label for this new subject is predicted as i*.

**Remark 3.1** *The main difference between the MLR-HomoNet and LR-HeteNet methods is that the MLR-HomoNet method adopts the feature screening approach to retain informative predictors by pooling all subjects, while the feature screening approach of the LR-HeteNet method retains predictors under subjects that are in a specific class. It suggests that the estimated active sets* (19) *depend on the class and are different from each other, and thus, the resulting network structures determined by Step 2 of Algorithm 2 are different based on different classes. Therefore, we conclude that the MLR-HomoNet method only adopts different levels of gene expression values to classify tumor samples, while the LR-HeteNet method uses not only gene expression values but also class-dependent network structures to do the classification*.

## 4 Results

In this section, we aim to implement Algorithms 1 and 2 in Section 3 to the GCM dataset introduced in Section 2.1.

### 4.1 Detection of informative gene expressions via feature screening

In the GCM dataset, there are *I* = 14 classes. The dimension of predictors is *p* = 16, 063 and the sample size is *n* = 198, where the size of the training set is 144 and the size of the testing set is 54. Following steps in Fig 1, we first implement the proposed method in Section 3 to fit models based on the training set, and then assess the performance of prediction by examining the testing set.

Since the dimension of predictors is extremely larger than the sample size, i.e., *p* ≫ *n*, to determine the informative predictors, we adopt the screening signal (2) to retain informative gene expressions. The first strategy in Algorithm 1 is to apply (2) to evaluate the signal of *X*_•*k*_ and *Y* ∈ {1, ⋯, 14} and determine the estimated active set (3); the second consideration in Algorithm 2 is to calculate the signal of *X*_•*k*_ and *Y*^*i*^ for *i* = 1, ⋯, 14 and then obtain the estimated class-dependent active set (19). As suggested in [33, 35, 36], under the training set, we consider to retain $[\frac{144}{\text{log}(144)}]=29$ gene expression values for the MLR-HomoNet method and retain $\left[\frac{n_{i}}{\text{log}(n_{i})}\right]$ gene expression values with *i* = 1, ⋯, 14 for the LR-HeteNet method, where *n*_*i*_ is the sample size of class *i* summarized in Table 1.

### 4.2 Network-based classification models

**Fig 2:**
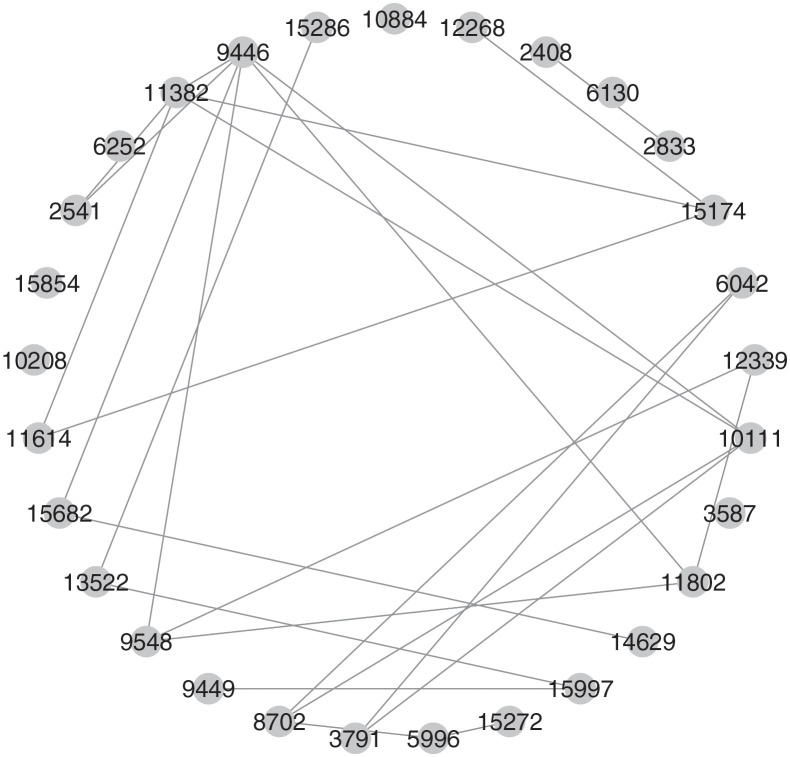
The whole network structure with selected gene expressions.

**Fig 3:**
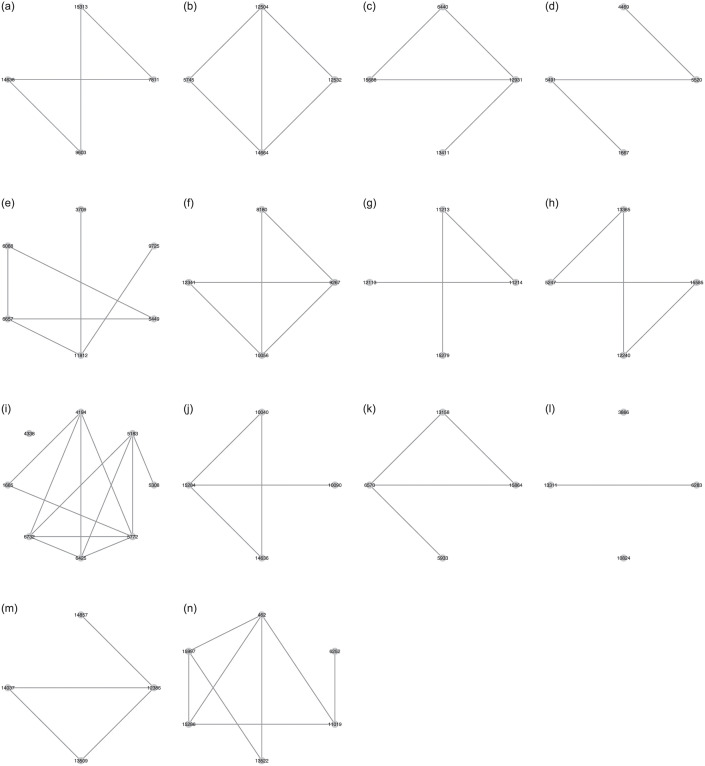
The network structure with selected gene expressions based on different cancers.

After the feature screening step, we next apply the estimation procedure in Section 3.2 to determine the network structure of selected gene expressions in the training set. Fig 2 displays the network structure with all samples accommodated, and the network structures of selected gene expressions based on different cancers are displayed in Fig 3. In Fig 2, we can see that the selected gene expressions have complex dependence structures. For example, gene expressions with ID 10111, 9548, and 9446 are connected with several gene expressions, while three gene expressions 10884, 15854, and 10208 have no connections with others. On the other hand, as shown in Fig 3, different classes have different selected gene expressions and associated network structures, which verifies the discussion in Remark 3.1. That is, as different kinds of cancer differ in their corresponding gene expressions, according to the specific network structures of gene expressions produced from our analysis, we can infer which cancer each tumor sample is from.

**Fig 4:**
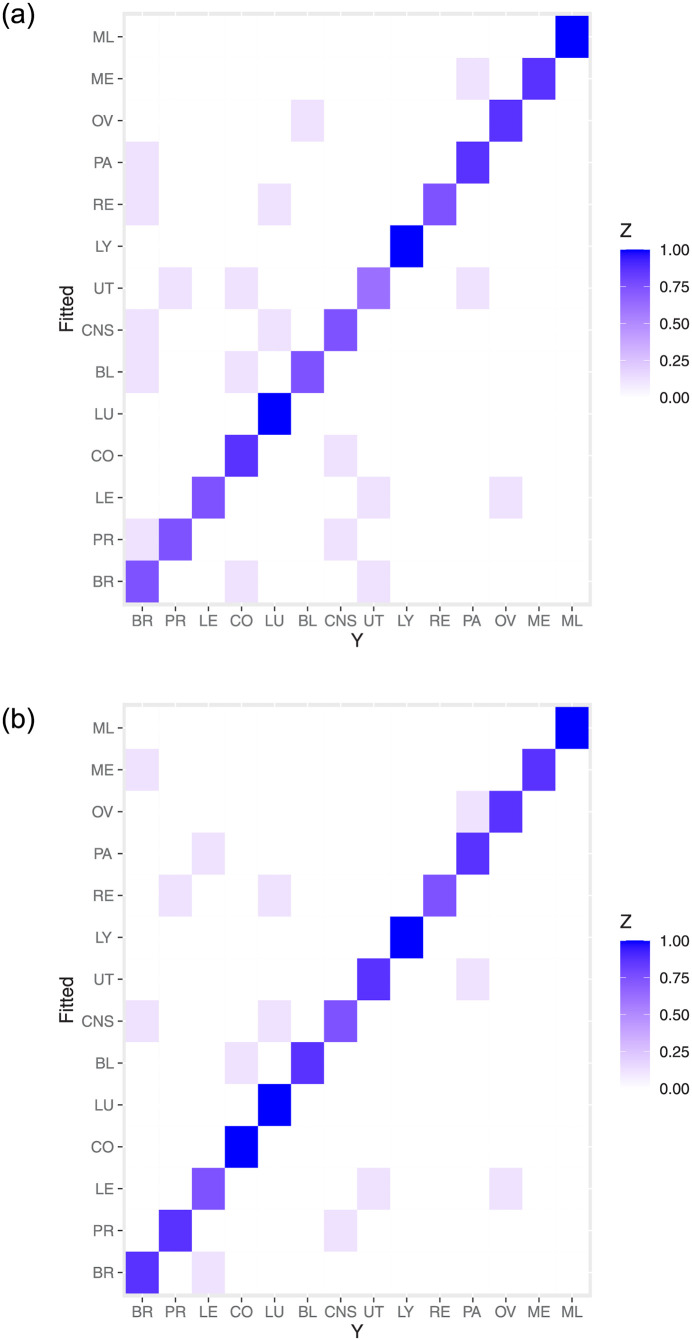
Heatmaps for the fitted values based on two proposed methods under the training data. The left panel is obtained by Algorithm 1, the right panel is obtained by Algorithm 2. *Z* represents the proportion of (mis)classification.

To adopt the determined network structures to examine the classification, we implement the network structures and the training set to the classification models proposed in Sections 3.3 and 3.4, respectively. To see the fitness of two models, we first implement the training data to the fitted models and examine the classification. The 14×14 confusion matrices based on the MLR-HomoNet and LR-HeteNet methods are shown in Tables 2 and 3, respectively, where columns are labels from the training data T, rows are labels of fitted values, diagonal entries reflect number of correct classification, and nondiagonal entries are number of misclassification by fitted values. In general, both methods show satisfactory model fittness as the accuracy of classification is high. Moreover, we observe that the LR-HeteNet method seems to slightly outperform the MLR-HomoNet method since the latter method produces slightly larger misclassification on BR, PR, CO, and UT than those of the former method. This result makes sense because the LR-HeteNet method is based on class-dependent network structure that can directly reflect the corresponding cancers. For a clear visualization, we further display two heatmaps in Fig 4, which are obtained by Tables 2 and 3 with each row divided by the class-dependent sample size in the training data. We observe that diagonal entries have dark color, which indicate that the proportion of true classification is high and Algorithms 1 and 2 give well-fitted models.

**Table 2:** A 14 × 14 confusion matrix: Model fittness based on the MLR-HomoNet method for the training data T.

	BR	PR	LE	CO	LU	BL	CNS	UT	LY	RE	PA	OV	ME	ML
BR	6	1	0	0	0	1	1	0	0	1	1	0	0	0
PR	0	6	0	0	0	0	0	1	0	0	0	0	0	0
LE	0	0	6	0	0	0	0	0	0	0	0	0	0	0
CO	1	0	0	7	0	1	0	1	0	0	0	0	0	0
LU	0	0	0	0	16	0	1	0	0	1	0	0	0	0
BL	0	0	0	0	0	6	0	0	0	0	0	1	0	0
CNS	0	1	0	1	0	0	6	0	0	0	0	0	0	0
UT	1	0	1	0	0	0	0	5	0	0	0	0	0	0
LY	0	0	0	0	0	0	0	0	24	0	0	0	0	0
RE	0	0	0	0	0	0	0	0	0	6	0	0	0	0
PA	0	0	0	0	0	0	0	1	0	0	7	0	1	0
OV	0	0	1	0	0	0	0	0	0	0	0	7	0	0
ME	0	0	0	0	0	0	0	0	0	0	0	0	7	0
ML	0	0	0	0	0	0	0	0	0	0	0	0	0	16

**Table 3:** A 14 × 14 confusion matrix: Model fittness based on the LR-HeteNet method for the training data T.

	BR	PR	LE	CO	LU	BL	CNS	UT	LY	RE	PA	OV	ME	ML
BR	7	0	0	0	0	0	1	0	0	0	0	0	1	0
PR	0	7	0	0	0	0	0	0	0	1	0	0	0	0
LE	1	0	6	0	0	0	0	0	0	0	1	0	0	0
CO	0	0	0	8	0	1	0	0	0	0	0	0	0	0
LU	0	0	0	0	16	0	1	0	0	1	0	0	0	0
BL	0	0	0	0	0	7	0	0	0	0	0	0	0	0
CNS	0	1	0	0	0	0	6	0	0	0	0	0	0	0
UT	0	0	1	0	0	0	0	7	0	0	0	0	0	0
LY	0	0	0	0	0	0	0	0	24	0	0	0	0	0
RE	0	0	0	0	0	0	0	0	0	6	0	0	0	0
PA	0	0	0	0	0	0	0	1	0	0	7	1	0	0
OV	0	0	1	0	0	0	0	0	0	0	0	7	0	0
ME	0	0	0	0	0	0	0	0	0	0	0	0	7	0
ML	0	0	0	0	0	0	0	0	0	0	0	0	0	16

### 4.3 Prediction

**Fig 5:**
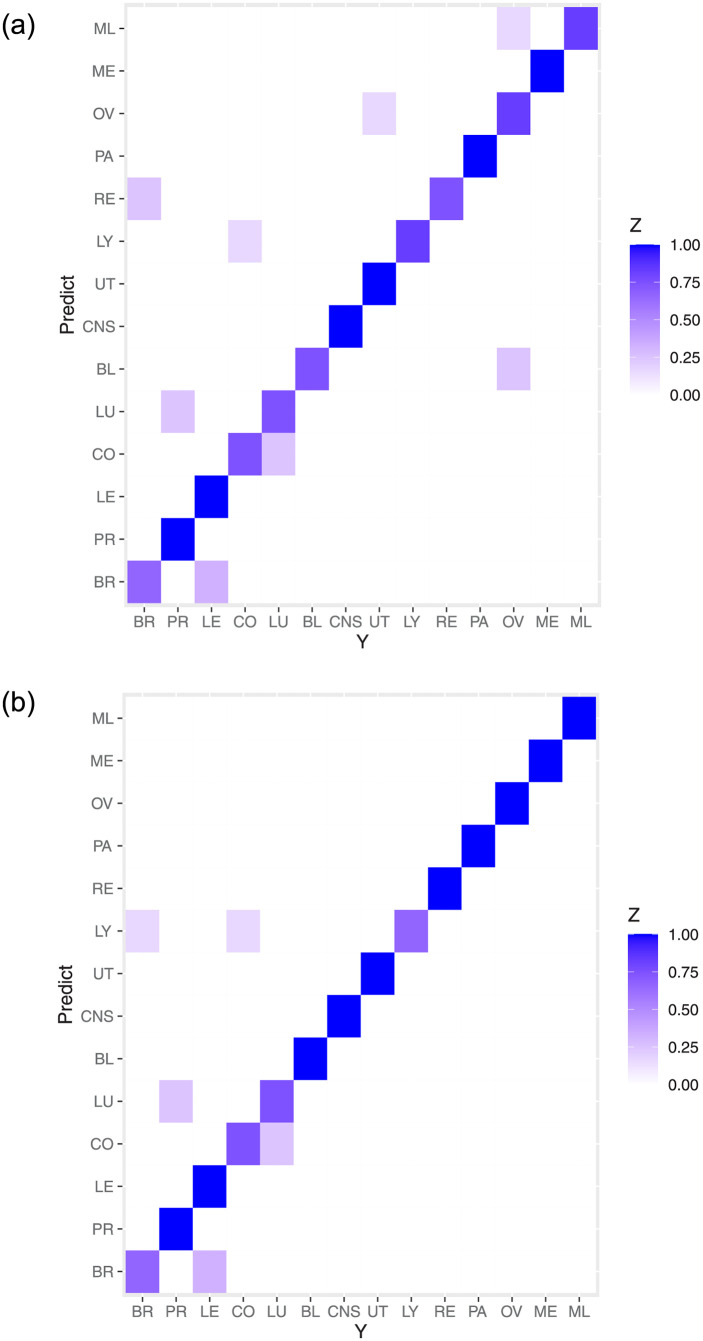
Heatmaps for the predicted values based on two proposed methods under the testing data. The left panel is obtained by Algorithm 1, the right panel is obtained by Algorithm 2. *Z* represents the proportion of (mis)classification.

When the predictive models are constructed, we now assess the performance of the proposed method by examining the prediction for the testing data. We implement the predictors in the testing data to the two proposed methods, and then make the prediction of classification. After that, we summarize the response in the testing data and the predictive classes to 14 × 14 confusion matrices in Tables 4 and 5, respectively, where columns are labels from the testing samples V, rows are labels of predicted values, diagonal entries reflect number of correct classification, and nondiagonal entries are number of misclassification by predicted values. Moreover, we also display two heatmaps in Fig 5 that are obtained by Tables 4 and 5 with each row divided by the class-dependent sample size in the testing data. From confusion matrices and heatmaps, We can see that two proposed methods have satisfactory performance in prediction because most of predicted classes are the same as class labels in the testing data, except for little misclassification.

**Table 4:** A 14 × 14 confusion matrix: Prediction based on the MLR-HomoNet method for the testing data V.

	BR	PR	LE	CO	LU	BL	CNS	UT	LY	RE	PA	OV	ME	ML
BR	2	0	0	0	0	0	0	0	0	1	0	0	0	0
PR	0	4	0	0	1	0	0	0	0	0	0	0	0	0
LE	1	0	2	0	0	0	0	0	0	0	0	0	0	0
CO	0	0	0	3	0	0	0	0	1	0	0	0	0	0
LU	0	0	0	1	3	0	0	0	0	0	0	0	0	0
BL	0	0	0	0	0	3	0	0	0	0	0	0	0	0
CNS	0	0	0	0	0	0	2	0	0	0	0	0	0	0
UT	0	0	0	0	0	0	0	3	0	0	0	1	0	0
LY	0	0	0	0	0	0	0	0	5	0	0	0	0	0
RE	0	0	0	0	0	0	0	0	0	3	0	0	0	0
PA	0	0	0	0	0	0	0	0	0	0	3	0	0	0
OV	0	0	0	0	0	1	0	0	0	0	0	5	0	1
ME	0	0	0	0	0	0	0	0	0	0	0	0	3	0
ML	0	0	0	0	0	0	0	0	0	0	0	0	0	5

**Table 5:** A 14 × 14 confusion matrix: Prediction based on the LR-HeteNet method for the testing data V.

	BR	PR	LE	CO	LU	BL	CNS	UT	LY	RE	PA	OV	ME	ML
BR	2	0	0	0	0	0	0	0	1	0	0	0	0	0
PR	0	4	0	0	1	0	0	0	0	0	0	0	0	0
LE	1	0	2	0	0	0	0	0	0	0	0	0	0	0
CO	0	0	0	3	0	0	0	0	1	0	0	0	0	0
LU	0	0	0	1	3	0	0	0	0	0	0	0	0	0
BL	0	0	0	0	0	4	0	0	0	0	0	0	0	0
CNS	0	0	0	0	0	0	2	0	0	0	0	0	0	0
UT	0	0	0	0	0	0	0	3	0	0	0	0	0	0
LY	0	0	0	0	0	0	0	0	4	0	0	0	0	0
RE	0	0	0	0	0	0	0	0	0	4	0	0	0	0
PA	0	0	0	0	0	0	0	0	0	0	3	0	0	0
OV	0	0	0	0	0	0	0	0	0	0	0	6	0	0
ME	0	0	0	0	0	0	0	0	0	0	0	0	3	0
ML	0	0	0	0	0	0	0	0	0	0	0	0	0	6

To assess the performance of classification and prediction numerically, we evaluate some commonly used criteria: micro averaged metrics, macro averaged metrics, and the adjusted Rand index. For a subject *j* in the testing data with *j* = 1, ⋯, 54, let ${\hat{y}}_{\text{new},j}$ denote the predicted class label determined by the prediction models and let *y*_new,*j*_ denote the class label in the testing data. For class *i* = 1, ⋯, *I*, we respectively calculate the number of the true positives (TP), the number of the false positives (FP), and the number of the false negatives (FN) as

$$\begin{array}{c} {\text{TP}}_{i}=\sum_{j=1}^{54}I(y_{\text{new},j}=i,{\hat{y}}_{\text{new},j}=i), \end{array}$$ (24)

$$\begin{array}{c} {\text{FP}}_{i}=\sum_{j=1}^{54}I(y_{\text{new},j}\neq i,{\hat{y}}_{\text{new},j}=i), \end{array}$$ (25)

and

$$\begin{array}{c} {\text{FN}}_{i}=\sum_{j=1}^{54}I(y_{\text{new},j}=i,{\hat{y}}_{\text{new},j}\neq i). \end{array}$$ (26)

For micro averaged metrics, precision and recall are, respectively, defined in terms of (24), (25), and (26):

$$\begin{array}{c} {\text{PRE}}_{\text{micro}}=\frac{\sum_{i=1}^{I}{\text{TP}}_{i}}{\sum_{i=1}^{I}{\text{TP}}_{i}+\sum_{i=1}^{I}{\text{FP}}_{i}} \end{array}$$ (27)

and

$$\begin{array}{c} {\text{REC}}_{\text{micro}}=\frac{\sum_{i=1}^{I}{\text{TP}}_{i}}{\sum_{i=1}^{I}{\text{TP}}_{i}+\sum_{i=1}^{I}{\text{FN}}_{i}}. \end{array}$$ (28)

Then Micro-F-score is defined as

$$\begin{array}{c} {\text{F}}_{\text{micro}}=2\times \frac{{\text{PRE}}_{\text{micro}}\times {\text{REC}}_{\text{micro}}}{{\text{PRE}}_{\text{micro}}+{\text{REC}}_{\text{micro}}}. \end{array}$$ (29)

On the other hand, for macro averaged metrics, for *i* = 1, ⋯, *I*, let ${\text{PRE}}_{i}=\frac{{\text{TP}}_{i}}{{\text{TP}}_{i}+{\text{FP}}_{i}}$ denote precision for class *i*, and let ${\text{REC}}_{i}=\frac{{\text{TP}}_{i}}{{\text{TP}}_{i}+{\text{FN}}_{i}}$ denote recall for class *i*. Then the overall precision and recall are, respectively, given by

$$\begin{array}{c} {\text{PRE}}_{\text{macro}}=\frac{1}{I}\sum_{i=1}^{I}{\text{PRE}}_{i} \end{array}$$ (30)

and

$$\begin{array}{c} {\text{REC}}_{\text{macro}}=\frac{1}{I}\sum_{i=1}^{I}{\text{REC}}_{i}; \end{array}$$ (31)

and Macro-F-score is defined as

$$\begin{array}{c} {\text{F}}_{\text{macro}}=2\times \frac{{\text{PRE}}_{\text{macro}}\times {\text{REC}}_{\text{macro}}}{{\text{PRE}}_{\text{macro}}+{\text{REC}}_{\text{macro}}}. \end{array}$$ (32)

According to the definitions, when all subjects are correctly classified, then FP and FN are equal to zero, yielding that PRE and REC are equal to one; if all subjects are falsely classified, then TP is equal to zero, and thus, PRE and REC are equal to zero. Therefore, values of PRE and REC are between zero to one. Moreover, the F-score falls in [0, 1] as well by treating 0/0 as zero. In principle, higher values of PRE, REC and F-score based on both micro and macro reflect better performance of methods ([20–22]).

In addition to criteria above, the other commonly used criterion is the adjusted Rand index (ARI). For *i*, *l* = 1, ⋯, *I*, let $n_{il}=\sum_{j=1}^{n}I(y_{\text{new},j}=i,{\hat{y}}_{\text{new},j}=l)$. Moreover, define $a_{i}=\sum_{l=1}^{I}n_{il}$ for *i* = 1, ⋯, *I* and $b_{l}=\sum_{i=1}^{I}n_{il}$ for *l* = 1, ⋯, *I*. Then ARI is defined as (e.g., [43])

ARI=∑i,l=1(nil2)-{∑i(ai2)∑l(bl2)}/(n2){∑i(ai2)+∑l(bl2)}/2-{∑i(ai2)∑l(bl2)}/(n2). (33)

As mentioned in [43], ARI is bounded above by one, and higher value of ARI indicates accurate classification.

We primarily adopt (27), (28), (29), (30), (31), (32), and (33) to assess the performance of two proposed methods. In addition, to compare with the proposed methods, we also examine several well established supervised learning methods, including logistic regression models *without* incorporating network structure [42], the support vector machine (SVM) that was examined by [30], K-nearest neighbor (KNN), linear discriminant analysis (LDA), Bayes, artificial neural network (ANN), XGBoost, random forest (RF), bagging, and long short-term memory (LSTM) methods. The implementation of corresponding R packages is summarized in Table 6.

**Table 6:** A list of existing methods and corresponding packages.

Method[Reference]	Function	R Package
SVM [44]	svm	e1071
KNN [45]	kNN	DMwR
LDA [46]	lda	MASS
Bayes [44]	naiveBayes	e1071
ANN [47]	neuralnet	neuralnet
XGBoost [48]	xgb.train	xgboost
RF [49]	randomForest	randomForest
Bagging [50]	ipredbagg	ipred
LSTM [51]	trainr	rnn

The prediction results of the proposed and competitive methods are summarized in Table 7. In general, we can observe that the two proposed methods have the largest values of PRE, REC, F-score, and ARI than other existing methods. For the comparisons among existing methods, we can see that advanced machine learning or deep learning methods (e.g., ANN, RF, Bagging) outperform the conventional ones, such as LDA or SVM, but are less satisfactory than the proposed methods because of slightly large misclassification. It verifies that incorporating network structures would improve the accuracy of classification and prediction. In addition, the other reason is that, unlike existing methods that possibly incur overfitting because of direct implementation of all gene expression values to fit models, the two proposed methods simply retain gene expression values and detect network structures that are related to the response, yielding parsimonious models. In this way, noises and impacts induced by irrelevant gene expression values can be eliminated. Compared with two proposed methods, we can see that the LR-HeteNet method outperforms the MLR-HomoNet method with larger values of criteria. The main reason is that the MLR-HomoNet model in Section 3.3 directly deals with multi-label classification by using a common network structure to classify tumors to the corresponding cancers. To simultaneously reflect information to all classes, the network structure displayed in Fig 2 is expected to require more gene expression values and complex interactions. On the other hand, the LR-HeteNet method in Section 3.4 identifies predictors and unique network structure to reflect a specific cancer, suggesting that types of cancers can be uniquely represented by different network structures of gene expression values. As shown in Fig 3, one can directly adopt a given network structure to classify tumors to their cancers with high accuracy of prediction. In summary, with noise induced by irrelevant predictors removed and informative network structures of predictors accommodated, the accuracy of classification and prediction has significant improvement.

**Table 7:** Prediction of classification for the testing data V.

Method	PRE_micro_	REC_micro_	F_micro_	PRE_macro_	REC_macro_	F_macro_	ARI
Agresti	0.693	0.697	0.695	0.688	0.696	0.692	0.453
SVM	0.801	0.812	0.806	0.813	0.820	0.816	0.786
LDA	0.705	0.705	0.705	0.699	0.694	0.696	0.474
KNN	0.677	0.663	0.670	0.654	0.666	0.660	0.433
Bayes	0.837	0.838	0.838	0.840	0.838	0.839	0.804
ANN	0.844	0.845	0.844	0.844	0.844	0.844	0.821
XGBoost	0.816	0.818	0.817	0.820	0.816	0.818	0.797
RF	0.840	0.838	0.839	0.842	0.841	0.841	0.813
Bagging	0.840	0.836	0.838	0.841	0.841	0.841	0.809
LSTM	0.835	0.837	0.836	0.837	0.840	0.838	0.794
MLR-HomoNet	0.856	0.871	0.863	0.867	0.878	0.872	0.833
LR-HeteNet	0.884	0.896	0.890	0.903	0.910	0.906	0.856

## 5 Discussion

In this paper, we present the network-based classification method to predict the classification of the tumor samples, which is an ultrahigh dimensional system, i.e., with multitudinous gene expressions as predictors. In the proposed method, we first adopt model-free feature screening technique to retain informative gene expressions from ultrahigh-dimensional data. After that, we identify the network structures of the detected gene expressions based on different cancers, and the property of the network structure recovery allows us to fit the nominal logistic regression based on the network structure and examine the classification and prediction. Compared with other existing methods, the proposed method gives more precise prediction results.

There are several possible extensions based on the current work. For example, the RNA sequences, regarded as count data, are also frequently explored in bioinformatics. The proposed method can be naturally extended to deal with the RNA sequence data by treating them as the predictors because the signal of detecting predictors (2) is free of distribution of random variables, and the identification of network structure in Section 3.2 is based on exponential family graphical models. For the implementation of classification models, it is interesting to explore other machine learning methods, such as SVM, LDA, or KNN, and other deep learning approaches that are popular in data science.

Moreover, the research gap still exists and more explorations can be done by extending the proposed method. For example, as discussed in [32], measurement error in predictors is ubiquitous in data analysis, especially that mismeasurement is inevitable in gene expression data (e.g, [52]). Ignoring measurement error effects is expected to increase the possibility of false classification and lead to wrong conclusion. Therefore, it is important to develop a new error-eliminating strategy to deal with measurement error based on the current method. Finally, as R packages associated with some of the existing methods have been developed, the new method proposed here anticipates a corresponding R package.

## Supporting information

S1 Data (ZIP)
